# Internet Use and Psychosomatic Symptoms among University Students: Cross-Sectional Study

**DOI:** 10.3390/ijerph19031774

**Published:** 2022-02-04

**Authors:** Gregor Stiglic, Ruth Masterson Creber, Leona Cilar Budler

**Affiliations:** 1 Faculty of Health Sciences, University of Maribor, 2000 Maribor, Slovenia; leona.cilar1@um.si; 2 Faculty of Electrical Engineering and Computer Science, University of Maribor, 2000 Maribor, Slovenia; 3 Usher Institute, University of Edinburgh, Edinburgh EH8 9YL, UK; 4 Department of Population Health Sciences, Division of Health Informatics, Weill Cornell Medicine, New York, NY 10065, USA; rmc2009@med.cornell.edu

**Keywords:** psychological symptoms, somatic symptoms, technology use, wellbeing, university students

## Abstract

Background: Although the internet facilitates access to a wide range of knowledge and evidence, overuse among young people is associated with lower wellbeing and psychosomatic symptoms. The aim of this cross-sectional study is to explore the relationship between internet use, mental wellbeing, and psychosomatic symptoms among university students in Slovenia. Methods: We used correlation matrix plots to identify correlated symptoms and multivariate logistic regression to analyze the relationship between the time spent on the internet or computer and psychosomatic symptoms controlling for gender. Symptoms were measured using the Health Behavior of School Children scale. Results: Out of 464 students, the majority (64.7%, *n* = 300) were healthcare students and 35.3% (*n* = 164) were computer science students. Among somatic symptoms, headaches were associated with more time spent on the computer (r = −0.17, *p* < 0.001) and were significantly more prevalent in computer science students compared to health science students (χ2(1) = 8.52, *p* = 0.004). Time spent using the internet for spare time activities was associated with lower nervousness (r = 0.15, *p* = 0.005). Conclusions: Computer science students reported more frequent psychological symptoms compared to health science students and less somatic symptoms.

## 1. Introduction

There are over 4 billion regular internet users globally, and the largest growth rate is among young adults. Developments in information technology have positive effects on different aspects of human life [1]. The Internet has become the main source of health-related information due to the high-speed search, low costs, and the ability to access information anonymously [2]. However, there has been much more awareness about the adverse consequences of the overuse of the internet and social media on student’s mental health and mental wellbeing, academic achievement [3,4,5], dietary habits, sleep problems and fatigue symptoms [6,7], and internet addiction [8,9].

Mental health problems among university students are highly prevalent [10,11,12,13,14,15] and are estimated to affect between 12–48% of students [16]. In the context of COVID-19, university students are almost completely dependent on the internet for academic purposes, entertainment, and interpersonal communication [17,18].

Overuse of the internet can exacerbate depression, anxiety, and stress for students across a variety of fields of study [19,20], including both students in schools of engineering and nursing. A study conducted among engineering students in India reported that internet addiction was associated with psychosomatic symptoms, including fatigue, insomnia, headache, eating disorders, etc. [17]. Internet technology is infused in almost all study aspects of engineering student and impact their health, behavior, academic success, and job performance [21]. Anand et al. [22] emphasized the need for early identification and management of internet addictions among engineering students. Consistent with this finding, Khalil et al. [23] reported a positive association between higher internet use and increased psychosomatic symptoms, social appearance anxiety, and nomophobia—fear of being detached from a smartphone—among nursing students [24]. Another study [25] revealed that older nursing students were more prone to excessive internet use than younger nursing students. In addition, students in which problematic internet use was detected managed their time poorer.

Students’ awareness about the positive and negative impacts of the internet is important for safe internet use. There is a need to investigate the relationship between internet and computer use and psychosomatic symptoms among university students to propose educational and supportive interventions for students. Psychosomatic disorder can impact learning skills and academic success, leading to worsening students’ mental wellbeing and study satisfaction. The aim of this study was to investigate the relationship between internet and computer use and psychosomatic symptoms among health sciences and engineering students.

## 2. Materials and Methods

Data for this cross-sectional study were collected using an anonymous online survey at two faculties belonging to a single university in Slovenia from January until March 2019 (pre and post examination period) using convenience sampling. Participants in the study did not receive any financial compensation. All data is available in Supplementary Materials (File S1).

### 2.1. Participants

A total of 464 students from a university in Slovenia were enrolled in the study. The health sciences sample included 300 students (64.7%) who were earning degrees in Nursing, Bioinformatics, and Management in Health and Social Organization. The engineering sample included 164 (35.3%) students who were earning degrees in Electrical Engineering and Computer Science. Students were invited to participate in the study by university teachers and teaching assistants during different classes. The decision to take part in the study was voluntary. The age range of the involved students ranged from 18 to 49, with a mean of 21.69 (SD = 4.38). Other demographic characteristics of the sample are shown in Table 1.

### 2.2. Measures

We used a validated and translated version of the Warwick–Edinburgh Mental Wellbeing-Scale (WEMWBS) questionnaire to measure students’ mental wellbeing [26,27,28]. The WEMWBS includes 14 items that measure positive mental wellbeing over the last two weeks, with responses that range from 1 (none of the time) to 5 (all of the time). A total WEMWBS score was calculated by summing all 14-item responses resulting in scores ranging from 14 to 70 [27]. The internal consistency calculated using Cronbach alpha on our sample was 0.902. 

Psychosomatic symptoms were measured using the Health Behavior of School Children (HBSC) scale and an additional question relating to self-assessment of health [29]. The HBSC symptom checklist includes eight common symptoms: headache, stomachache, backache, feeling low, irritability, bad-tempered, feeling nervous, difficulty in falling asleep, and feeling dizzy.

The eight symptoms were grouped into two groups: somatic (headache, stomachache, backache, feeling dizzy) and psychological (feeling low, irritability, bad-tempered, and feeling nervous) symptoms [22]. Participants self-reported how often they experienced symptoms, ranging from ‘about every day’ to ‘rarely or never’. The self-assessment of the answers ranged from 1 (poor) to 5 (excellent), with higher scores indicating the lower frequency of the specific symptom (and better self-rated health) [29]. The internal consistency of the HBSC scale for our sample was 0.780. Measures were chosen to include similar concepts and those related to mental wellbeing. Participants in the study were also asked about their time (in hours) spent on the internet for study and spare time activities. They were also asked to estimate their time using mobile phones and computers.

### 2.3. Data Analysis

All data analysis, including visualization of the data, was conducted using R statistical programing language (R Foundation for Statistical Computing, Vienna, Austria, 2005) [30,31]. The response values from psychosomatic Likert scale questions were summed to obtain the total scores for somatic, psychological, and psychosomatic symptoms. Since both somatic and psychological symptoms contain four items, the maximum score in both scales was 20 points and minimal 5 points where a higher value represents the lower frequency of symptoms. In the self-assessment of health questions in the HBSC, the answers ranged from 1 (poor) to 5 (excellent). Most of the answers were mandatory to complete resulting in a very low rate of missing data (less than 1% in any variable). To account for missing data in the regression analysis, we used imputation of the missing values using the missForest package in R [32].

**Figure 1:**
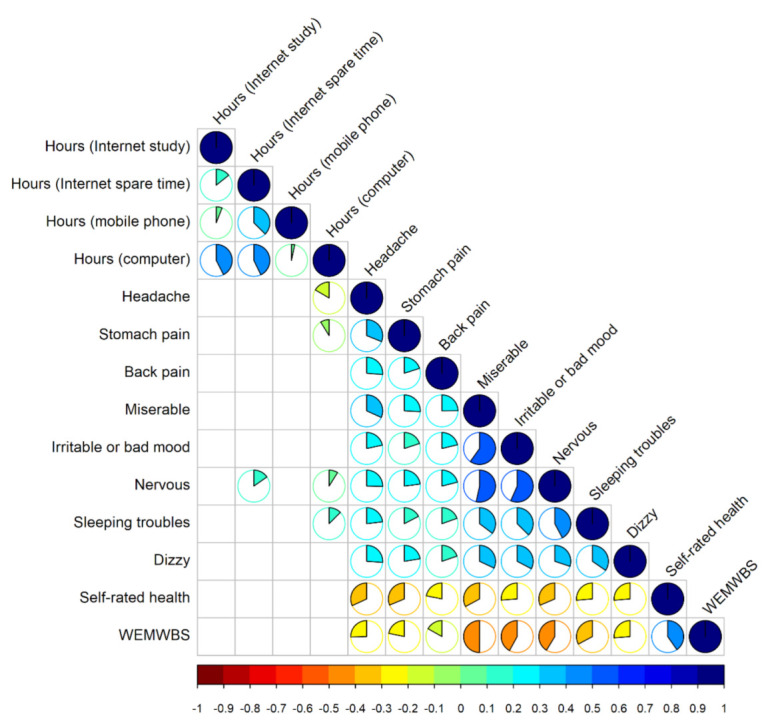
Bivariate correlations between four measures of internet use (study or spare time), mobile phones and computer use, eight psychosomatic symptoms, self-rated health, and mental-wellbeing (WEMWBS) scores.

Results for the correlation matrix visualization were calculated using the Spearman regression coefficient as implemented in the corrplot R package [33] using only complete (pairwise) samples. Additionally, we used multiple linear regression to analyze the partial correlation coefficients between time spent on the internet, on mobile phones, or computers and psychological or somatic symptoms controlling for gender. Variance inflation factor calculations were performed to examine potentially problematic multicollinearity among predictors with values below 10 considered as acceptable [34]. Figure 1 show significant correlations where the Spearman correlation test resulted in a statistically significant result (α = 0.05). Differences between computer science and health science students were assessed using the chi-square test. For measures that were highly positively skewed, we used square root transformation to obtain normal data distribution.

## 3. Results

The correlation matrix (Figure 1) show bivariate correlations between all four measures of time spent on the internet (study or spare time), computer, or mobile phone and eight psychosomatic symptoms. Hours refer to hours per day used for various activities, i.e., hours per day for internet use for study purposes. As demonstrated in Figure 1, there was a strong correlation between Hours (computer), Hours (Internet spare time), and Hours (mobile phone). Additionally, self-rated health and WEMWBS scores were also included in the exploratory correlation analysis. Interestingly, no statistically significant correlation could be demonstrated between WEMWBS or self-rated health and hours spent using a computer, the internet, or a mobile phone. Feeling nervous was the only symptom that was correlated with the time spent on the internet in spare time (r = 0.153, 95% CI [0.039, 0.218], *p* = 0.005). There were two somatic and two psychological symptoms correlated with the number of hours spent on the computer. Headache (r = −0.166, 95% CI [−0.256, −0.079], *p* < 0.001) and stomach pain (r = −0.088, 95% CI [−0.185, −0.005], *p* = 0.039) were both positively correlated with computer time. On the other hand, feeling nervous (r = 0.089, 95% CI [0.016, 0.196], *p* = 0.021) and sleeping troubles (r = 0.125, 95% CI [0.005, 0.186], *p* = 0.039) had a negative correlation with computer time.

The multiple regression analysis controlled gender and included two sets of four regression models. Associations between the time spent on the internet (study or spare time), mobile phone, or computer and somatic symptoms (headache, stomach pain, back pain, feeling dizzy) were identified.

For somatic symptoms, feeling dizzy was correlated (β = 0.068, *p* = 0.046) with hours of time spent on the internet for study. Headaches were also negatively correlated (β = −0.080, *p* = 0.037) with hours spent on a computer (Table 2). No significant contribution of the psychological symptoms was observed in any of the four models (Table 3).

**Figure 2:**
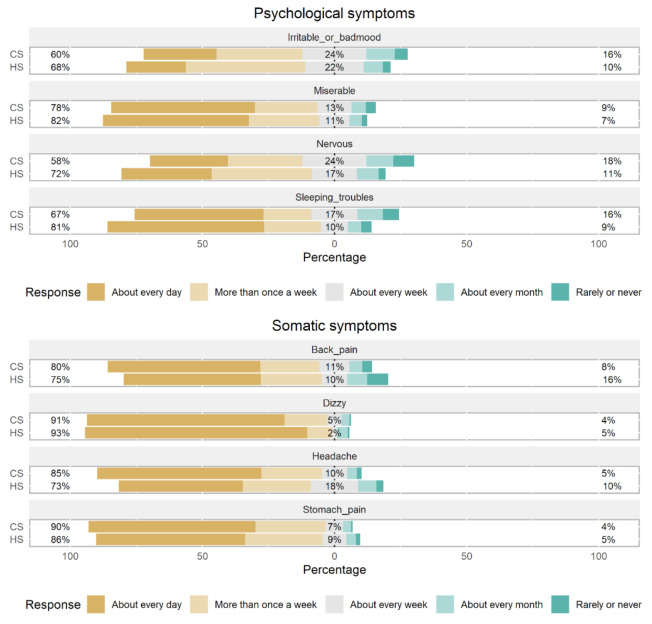
Distribution of responses representing the self-reported frequency of psychological and somatic symptoms for computer science (CS) and health science (HS) students.

To confirm the assumption that there are significant differences in reported psychosomatic symptoms between the students from computer science (CS) and health science (HS) schools, we compared all eight symptoms by faculty. Likert scale plots were used to visually demonstrate the differences in the distribution of the responses among the two observed groups. Figure 2 show two sets of four symptoms demonstrating a higher frequency of somatic and lower frequency of psychological symptoms reported by the CS students compared to HS students.

Generally, we can observe a very low frequency of answers representing a low level of reported symptoms, ranging from 4% to 18% (Figure 2). Due to high skewness in the distribution of the results, we grouped Likert scale results in two categories—“more than once per week” and “about every week or less”. Feeling nervous (χ^2^(1) = 9.50, *p* = 0.002) and sleeping troubles (χ^2^(1) = 10.60, *p* = 0.001) were the two psychological symptoms with statistically significant differences between CS and HS students. On the other hand, for somatic symptoms, CS students reported much more frequent headaches compared to HS students (χ^2^(1) = 8.52, *p* = 0.004).

## 4. Discussion

The purpose of this study was to investigate the relationship between internet and computer use and psychosomatic symptoms among university students. Our results show a weak correlation between time spent on the internet and psychological symptoms and a stronger association with somatic symptoms, including both headaches and stomachaches with computer time.

Young people, especially students, are at higher risk of experiencing negative psychosomatic symptoms, and attention should be given to the detention and treatment of those symptoms [35]. Computer Vision Syndrome (CVS) symptoms are defined as “a complex of eye and vision problems resulting from the activities which stress the near vision during the use of the computers and digital screens.” [36]. The excessive use of digital equipment is a risk factor for migraine-type development [37]. A study among school-aged children showed positive correlations between computer use, stomach pain, and school computer-related musculoskeletal outcomes [38]. Another study showed that frequency of headaches was positively correlated with weekly hours of computer use, frequency, and duration of usage, and no correlation between stomach pain and computer usage [39].

In our study, students who spent more time using the internet in their spare time reported less nervousness, with no correlation found between internet use for study and nervousness. One reason for this may be that students who are using the internet in their spare time are using it to connect with friends, family, and for social interactions. These study findings are in contrast with those reported among adolescents in Finland, France, and Denmark, where computer use was associated with shorter sleep duration and higher symptom load [40]. A difference between the studies is that we differentiated between overall internet usage and internet use in spare time versus internet use for study versus spare time. Thomee and colleagues also found that extreme computer usage in both men and women can cause loss of sleep, depression, and other serious mental health issues [41]. Poor sleep quality and insomnia are both common among university students and more prevalent among nocturnal computer users. Moreover, headaches associated with computer use also deteriorate the quantity and quality of sleeping [42]. Contrary to the recent findings [19,20], our results did not show any statistically significant correlation between WEMWBS or self-rated health and hours spent using a computer, the internet, or a mobile phone.

It is evident that there are significant differences in reported psychosomatic symptoms between the students from computer science and health science. We did not explore the reasons for this; thus, this is one of the study limitations. The differences are shown in a higher frequency of somatic (headache, stomachache, backache, feeling dizzy) and lower frequency of psychological (feeling low, irritability, bad-tempered, and feeling nervous) symptoms among computer science students compared to health science students. This may be due to the fact that computer science students spend more time using a computer. More time spent using a computer is related to more frequent stomach aches, irritability, feeling nervous, and difficulty falling asleep [43]. Moreover, nursing students experience various psychological symptoms, such as preoccupation with danger, hopelessness, self-blame, and report lower psychological wellbeing [44].

Given the growing problem of internet addiction studied by many scientists and mental health professionals, this work represents an important contribution to the body of knowledge that could be used in designing mental wellbeing programs for students. Such programs could be tailored to specific groups of students based on their type of study, year of study, and study program. Students need to be comprehensively educated about internet addiction and the harms that overuse of screens and social media can have on their physical and mental wellbeing [45]. Other than training, counseling services should be available for all university students [23].

The main limitations in this study are related to the distribution of different subgroups in the population. For example, an uneven distribution of gender in CS and HS populations did not allow representative comparison by gender in both schools. Another sample-based limitation relates to the generalizability of the findings based on the young age of participants; 88% of the students enrolled in both schools belonged to the age group of 18–25 years. Self-reported hours of internet, computer, or phone use were also highly skewed with some potential outliers where it was difficult to assess the validity of the data. To reduce the impact of this limitation, all four variables were transformed using square root transformation. Since all measures of internet, computer, and phone usage were self-reported, they might not represent an objective measure compared to some studies where different ways of measuring the time spent on a computer or the internet were used.

## 5. Conclusions

Internet use has both positive and negative impacts on psychosomatic symptoms among students. Internet use should be measured with granularity to parse out differences in the impact of using the internet for study versus in spare time. We report that students who were using the internet in their spare time reported less nervousness. We also report that more time on the computer was associated with more frequent headaches. We found that the prevalence of headaches was higher among computer science compared to health science students. Health science students did report a much higher frequency of sleeping troubles and nervousness.

## Figures and Tables

**Table 1:** Demographic characteristics of the sample by faculty.

	Health Sciences (*n* = 300)		Electrical Engineering and Computer Science (*n* = 164)	
	N	%	N	%
Gender				
Female	255	84.2	30	18.1
Male	48	15.8	136	81.9
Level of study				
1st (Undergraduate)	245	80.6	125	75.3
2nd (Master)	51	16.8	40	24.1
3rd (PhD)	8	2.6	1	0.6
Study year				
1st	128	42.1	152	92.1
2nd	81	26.6	13	7.9
3rd	95	31.3		
Study type				
Full time	226	74.3	165	99.4
Part time	78	25.7	1	0.6

**Table 2:** Regression coefficients for somatic symptoms models.

	Internet (Study)		Internet (Freetime)		Hours (Computer)		Hours (Phone)	
	Estimate	Std.	Estimate	Std.	Estimate	Std.	Estimate	Std.
(Intercept)	**1.378**	**0.072**	**1.490**	**0.083**	**1.323**	**0.107**	**1.784**	**0.114**
Female	**0.099**	**0.051**	**0.435**	**0.058**	**0.868**	**0.075**	−0.056	0.080
Headache	−0.041	0.026	0.010	0.030	**−0.080**	**0.039**	0.027	0.042
Stomach pain	−0.021	0.030	0.048	0.035	−0.013	0.044	0.060	0.048
Back pain	0.008	0.021	−0.028	0.024	0.012	0.031	−0.016	0.033
Dizzy	**0.068**	**0.034**	0.031	0.039	0.051	0.050	0.049	0.054

Statistically significant (α < 0.05) coefficient values are written in bold.

**Table 3:** Regression coefficients for psychological symptoms models.

	Internet (Study)		Internet (Freetime)		Hours (Computer)		Hours (Phone)	
	Estimate	Std.	Estimate	Std.	Estimate	Std.	Estimate	Std.
(Intercept)	**1.387**	**0.066**	**1.472**	**0.075**	**1.191**	**0.096**	**1.812**	**0.103**
Gender = Female	**0.117**	**0.050**	**0.420**	**0.057**	**0.877**	**0.074**	−0.080	0.079
Miserable	−0.014	0.034	0.049	0.038	−0.001	0.049	−0.085	0.053
Irritable or bad mood	−0.007	0.033	−0.027	0.037	−0.059	0.048	0.087	0.052
Nervous	0.012	0.029	0.055	0.033	0.071	0.042	0.054	0.045
Sleeping troubles	−0.001	0.024	−0.020	0.027	0.020	0.035	−0.005	0.038

Statistically significant (α < 0.05) coefficient values are written in bold.

## Data Availability

The data presented in this study are available in the Supplementary Material (S1_Data.sav).

## Supplementary Materials

The following supporting information can be downloaded at: https://www.mdpi.com/article/10.3390/ijerph19031774/s1, File S1: The data presented in this study (S1_Data.sav).

## References

[1] Shirazi F., Heidari S., Fard S.J., Ghodsbin F. (2019). Pattern of internet use by iranian nursing students. Facilitators and barriers. Investig. Y Educ. Enferm..

[2] Sharma S., Oli N., Thapa B. (2019). Electronic health–literacy skills among nursing students. Adv. Med. Educ. Pract..

[3] Feng S., Wong Y.K., Wong L.Y., Hossain L. (2019). The Internet and Facebook Usage on Academic Distraction of College Students. Comput. Educ..

[4] Gulati D.D.K., Bakliwal M. (2019). A Study of Internet Usage and Study Habits among Students.

[5] Azeems M.S. (2017). Use of social media and its perceived influence on the academic performance of university students. Anthropol. Bull..

[6] Bener A., Yildirim E., Torun P., Çatan F., Bolat E., Alıç S., Akyel S., Griffiths M.D. (2018). Internet Addiction, Fatigue, and Sleep Problems Among Adolescent Students: A Large-Scale Study. Int. J. Ment. Health Addict..

[7] Baturay M.H., Toker S. (2019). Internet addiction among college students: Some causes and effects. Educ. Inf. Technol..

[8] Young K.S. (2007). Cognitive Behavior Therapy with Internet Addicts: Treatment Outcomes and Implications. Cyberpsychol. Behav..

[9] Khamis S., Ahmad A., Ahmad M. (2019). A descriptive analytic model of internet usage and student performance. Technol. Manag..

[10] Lo S.M., Wong H.C., Lam C.Y., Shek D. (2018). Common Mental Health Challenges in a University Context in Hong Kong: A Study Based on a Review of Medical Records. Appl. Res. Qual. Life.

[11] McLafferty M., Lapsley C.R., Ennis E., Armour C., Murphy S., Bunting B.P., Bjourson A.J., Murray E.K., O’Neill S.M. (2017). Mental health, behavioural problems and treatment seeking among students commencing university in Northern Ireland. PLoS ONE.

[12] Bruffaerts R., Mortier P., Kiekens G., Auerbach R.P., Cuijpers P., Demyttenaere K., Green J., Nock M.K., Kessler R.C. (2017). Mental health problems in college freshmen: Prevalence and academic functioning. J. Affect. Disord..

[13] Pedrelli P., Nyer M., Yeung A., Zulauf C., Wilens T. (2014). College Students: Mental Health Problems and Treatment Considerations. Acad. Psychiatry.

[14] Harrer M., Adam S.H., Baumeister H., Cuijpers P., Karyotaki E., Auerbach R.P., Kessler R.C., Bruffaerts R., Berking M., Ebert D.D. (2018). Internet interventions for mental health in university students: A systematic review and meta-analysis. Int. J. Methods Psychiatr. Res..

[15] Cilar L., Barr O., Štiglic G., Pajnkihar M. (2019). Mental well-being among nursing students in Slovenia and Northern Ireland: A survey. Nurse Educ. Pract..

[16] Auerbach R.P., Mortier P., Bruffaerts R., Alonso J., Benjet C., Cuijpers P., Demyttenaere K., Ebert D.D., Green J.G., Hasking P. (2018). WHO World Mental Health Surveys International College Student Project: Prevalence and distribution of mental disorders. J. Abnorm. Psychol..

[17] Kumar R. (2014). Internet addiction and psychosomatic symptoms in engineering students. Delhi Psychiatry J..

[18] Harerimana A., Mtshali N.G. (2018). Internet usage among undergraduate nursing students: A case study of a selected university in South Africa. J. Nurs. Educ. Pract..

[19] Gupta A., Khan A.M., Rajoura O.P., Srivastava S. (2018). Internet addiction and its mental health correlates among undergraduate college students of a university in North India. J. Family Med. Prim. Care.

[20] Barthakur M., Sharma M. (2012). Problematic internet use and mental health problems. Asian J. Psychiatry.

[21] Bisen S.S., Deshpande Y., Haridas K. (2019). Does personality traits predict excessive use of internet technology among engineering students? Simple mediation analysis. Int. J. Electr. Eng. Educ..

[22] Anand N., Jain P.A., Prabhu S., Thomas C., Bhat A., Prathyusha P.V., Bhat S.U., Young K., Cherian A.V. (2018). Internet Use Patterns, Internet Addiction, and Psychological Distress Among Engineering University Students: A Study from India. Indian J. Psychol. Med..

[23] Khalil A.I., Alharbi N.B., Alhawasawi H.Y., Albander A.B. (2016). Prevalence of Internet Addiction among Nursing Students and the Association with their Academic Performance and Mental Health. Athens J. Health.

[24] Ayar D., Gerçeker G.., Özdemir E.Z., Bektaş M. (2018). The Effect of Problematic Internet Use, Social Appearance Anxiety, and Social Media Use on Nursing Students’ Nomophobia Levels. CIN Comput. Inform. Nurs..

[25] Öksüz E., Guvenc G., Mumcu S. (2018). Relationship between Problematic Internet Use and Time Management among Nursing Students. CIN Comput. Inform. Nurs..

[26] Taggart F., Friede T., Weich S., Clarke A., Johnson M., Stewart-Brown S. (2013). Cross Cultural Evaluation of the Warwick-Edinburgh Mental Well-Being Scale (WEMWBS)—A Mixed Methods Study. Health Qual. Life Outcomes.

[27] Taggart F., Stewart-Brown S., Parkinson J. (2015). Warwick-Edinburgh Mental Well-Being Scale (WEMWBS): User Guide—Version 2.

[28] Tennant R., Hiller L., Fishwick R., Platt S., Joseph S., Weich S., Parkinson J., Secker J., Stewart-Brown S. (2007). The Warwick-Edinburgh mental well-being scale (WEMWBS): Development and UK validation. Health Qual. Life Outcomes.

[29] Currie C., van der Sluijs W., Whitehead R., Currie D., Rhodes G., Neville F., Inchley J. (2015). Findings from the HSBC 2014 Survey in Scotland: Health Behaviour in School-Aged Children, World Health Organization Collaborative Cross-National Study (HSBC).

[30] Stiglic G., Watson R., Cilar L. (2019). R you ready? Using the R programme for statistical analysis and graphics. Res. Nurs. Health.

[31] R Development Core Team (2005). A Language and Environment for Statistical Computing.

[32] Stekhoven D.J., Bühlmann P. (2012). MissForest—non-parametric missing value imputation for mixed-type data. Bioinformatics.

[33] Wei T., Simko V., Levy M., Xie Y., Jin Y., Zemla J. (2017). Package ‘corrplot’. Statistician.

[34] Cohen J., Cohen P., West S.G., Aiken L.S. (2013). Applied Multiple Regression/Correlation Analysis for the Behavioral Sciences.

[35] Akinbinu T.R., Mashalla Y.J. (2014). Impact of computer technology on health: Computer Vision Syndrome (CVS). Med. Pract. Preview.

[36] Iqbal M., El-Massry A., Elagouz M., Elzembely H. (2018). Computer Vision Syndrome Survey among the Medical Students in Sohag University Hospital, Egypt. Ophthalmol. Res. Int. J..

[37] Xavier M.K., Pitangui A.C., Silva G.R., Oliveira V.M., Beltrão N.B., Araújo R.C. (2015). Prevalence of headache in adolescents and association with use of computer and videogames. Cienc. Saude Coletiva.

[38] Harris C., Straker L., Smith A., Pollock C. (2012). A proposed model representing the relationships between user characteristics, computer exposure and musculoskeletal symptoms in children. Work.

[39] Harris C., Straker L., Pollock C., Smith A. (2015). Children, computer exposure and musculoskeletal outcomes: The development of pathway models for school and home computer-related musculoskeletal outcomes. Ergonomics.

[40] Nuutinen T., Roos E., Ray C., Villberg J., Välimaa R., Rasmussen M., Holstein B., Godeau E., Beck F., Léger D. (2014). Computer use, sleep duration and health symptoms: A cross-sectional study of 15-year olds in three countries. Int. J. Public Health.

[41] Thomée S., Härenstam A., Hagberg M. (2012). Computer use and stress, sleep disturbances, and symptoms of depression among young adults–a prospective cohort study. BMC Psychiatry.

[42] Salehi S.G., Hassani H., Mortezapour A., Sadeghniiat-Haghighi K. (2015). Assessing of sleepiness, insomnia and sleep quality among university students: Association between computer use and sleep quality. Ann. Mil. Health Sci. Res..

[43] Faridizad R., Ahadi Z., Heshmat R., Motlagh M.E., Sheidaei A., Ziaodini H., Taheri M., Qorbani M., Mahdavi S.B., Kelishadi R. (2019). Association of screen time with subjective health complaints in Iranian school-aged children and adolescents: The CASPIAN-V study. J. Public Health.

[44] Yüksel A., Bahadir-Yilmaz E. (2019). Relationship between depression, anxiety, cognitive distortions, and psychological well-being among nursing students. Perspect. Psychiatr. Care.

[45] Price A.M., Devis K., LeMoine G., Crouch S., South N., Hossain R. (2018). First year nursing students use of social media within education: Results of a survey. Nurse Educ. Today.

